# Marine fish traits follow fast-slow continuum across oceans

**DOI:** 10.1038/s41598-019-53998-2

**Published:** 2019-11-29

**Authors:** Esther Beukhof, Romain Frelat, Laurene Pecuchet, Aurore Maureaud, Tim Spaanheden Dencker, Jón Sólmundsson, Antonio Punzón, Raul Primicerio, Manuel Hidalgo, Christian Möllmann, Martin Lindegren

**Affiliations:** 1 Centre for Ocean Life, National Institute of Aquatic Resources (DTU Aqua), Technical University of Denmark, Kongens Lyngby, Denmark; 2 Institute of Marine Ecosystem and Fisheries Science, Center for Earth System Research and Sustainability (CEN), University of Hamburg, Hamburg, Germany; 3 Norwegian College of Fishery Science, UiT, The Arctic University of Norway, Tromsø, Norway; 4 Marine and Freshwater Research Institute, Reykjavik, Iceland; 5 Instituto Español de Oceanografía, Centro Oceanográfico de Santander, Santander, Spain; 6 Instituto Español de Oceanografía, Centre Oceanogràfic de les Balears, Ecosystem Oceanography Group (GRECO), Palma de Mallorca, Spain

**Keywords:** Fisheries, Biodiversity, Marine biology, Macroecology, Biogeography

## Abstract

A fundamental challenge in ecology is to understand why species are found where they are and predict where they are likely to occur in the future. Trait-based approaches may provide such understanding, because it is the traits and adaptations of species that determine which environments they can inhabit. It is therefore important to identify key traits that determine species distributions and investigate how these traits relate to the environment. Based on scientific bottom-trawl surveys of marine fish abundances and traits of >1,200 species, we investigate trait-environment relationships and project the trait composition of marine fish communities across the continental shelf seas of the Northern hemisphere. We show that traits related to growth, maturation and lifespan respond most strongly to the environment. This is reflected by a pronounced “fast-slow continuum” of fish life-histories, revealing that traits vary with temperature at large spatial scales, but also with depth and seasonality at more local scales. Our findings provide insight into the structure of marine fish communities and suggest that global warming will favour an expansion of fast-living species. Knowledge of the global and local drivers of trait distributions can thus be used to predict future responses of fish communities to environmental change.

## Introduction

Trait-environment relationships are crucial for predicting species and community responses to environmental change^1–3^. This is illustrated by a recent increase in large-scale studies on trait biogeography and its ties to the environment^4–6^. However, trait variation is typically expressed at an aggregated level of communities or functional groups in specific regions, or at a single and coarse spatial resolution, thereby not accounting for fine-scaled variability and potential scale-dependence in trait-environment relationships. These issues can now be addressed in marine ecosystem studies thanks to the increasing availability of comprehensive trait databases and species occurrence data across large areas^7,8^. Here, we compiled a high-resolution dataset of fish species abundances, environmental conditions and fishing pressure across the continental shelf seas of the North Atlantic and North-East Pacific, as well as trait information on size, growth, life history, reproduction and diet. Based on these data we investigated the trait biogeography of marine fish across environments using a set of complementary tools for big data analysis. These include a three-matrix ordination approach (RLQ analysis) and fourth-corner analysis, capable of summarizing spatial relationships between species abundances, traits, environmental conditions and fishing pressure^9^, and random forests (RFs), a machine learning technique that can model traits against environmental variables and allows projecting trait distributions based on the derived trait-environment relationships.

## Results and Discussion

**Figure 1:**
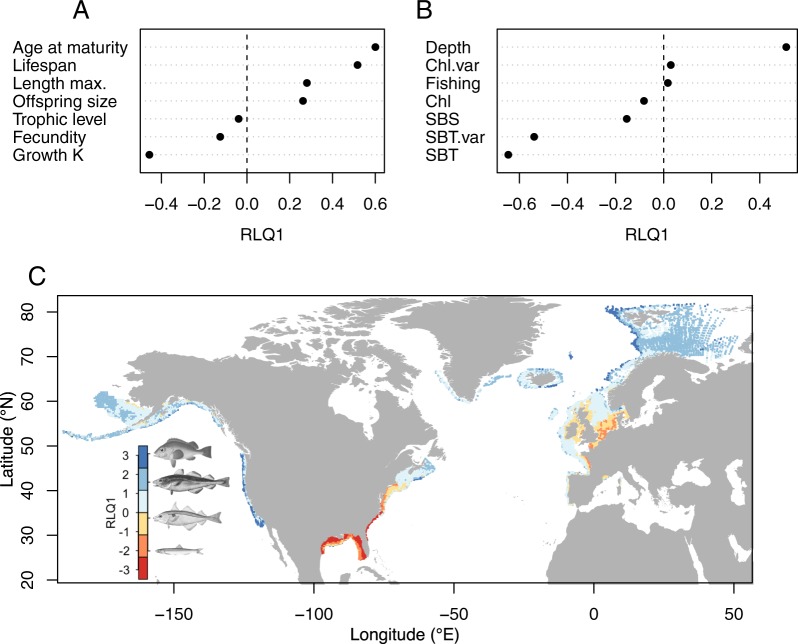
Results of the RLQ analysis summarizing the cross-covariance between traits and environmental variables across species and sampling sites. Scores of the traits (**A**), environmental variables (**B**), as well as the scores of all sampling sites and four examples of species on RLQ1 (**C**). The species range from having slow to fast life-history strategies and include (from top to bottom): golden redfish (*Sebastes norvegicus*), Atlantic cod (*Gadus morhua*), haddock (*Melanogrammus aeglefinus*) and European anchovy (*Engraulis encrasicolus*) (images derived from Couch^59^, NOAA Photo Library^60^, Goode^61^ and Gervais & Boulart^62^). Chl.var: seasonality in chlorophyll *a* concentration, Chl: chlorophyll *a* concentration, SBS: sea bottom salinity, SBT.var: seasonality in sea bottom temperature, SBT: sea bottom temperature.

The main relationships between traits and the environment are summarized by the first RLQ axis (RLQ1), explaining 97% of the cross-covariance between traits and environment across species and sampling sites (Fig. 1). The traits best represented by RLQ1 are the von Bertalanffy growth coefficient K, a proxy for individual growth rate, with highly negative scores, and age at maturity and lifespan with highly positive scores (Fig. 1A). Maximum body length, offspring size and fecundity are of intermediate importance, with only moderate scores on RLQ1, whereas trophic level has low importance with a score close to zero. The abiotic variables sea bottom temperature (SBT), seasonality in sea bottom temperature (SBT.var) and depth have high scores on RLQ1, while fishing pressure and seasonality in chlorophyll *a* concentration (Chl.var) show scores close to zero (Fig. 1B). Hence, areas with negative scores are characterized by high temperatures, strong seasonality and shallow depths, whereas areas with positive scores reflect deep waters with low temperatures and limited seasonality. These variables are thus the dominant environmental drivers determining the distribution of species conditioned on their traits.

**Figure 2:**
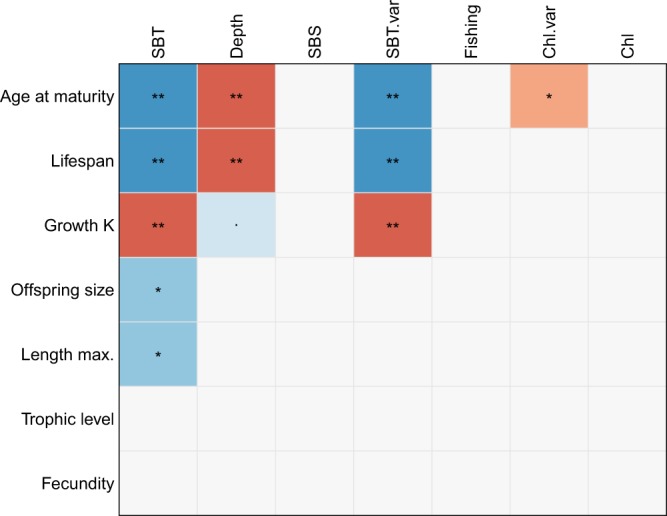
Fourth-corner analysis with 5,000 permutations demonstrating pairwise correlations between traits, environmental variables and fishing pressure. The p-values have been corrected for multiple comparison using false discovery rates. Cells in red indicate positive correlations and cells in blue negative correlations. The significance level is given by asterisks (‘•’: p-value < 0.1, ‘*’: < 0.05, ‘**’: < 0.01). Non-significant correlations are in light grey. SBT: sea bottom temperature, SBS: sea bottom salinity, Chl: chlorophyll *a* concentration, SBT.var: seasonality in sea bottom temperature, Chl.var: seasonality in chlorophyll *a* concentration.

The relationship between the environmental drivers and traits is also evident in the scores of the sampling sites on RLQ1, demonstrating a marked transition from negative to positive scores from lower to higher latitudes and from coastal to offshore waters along all three coastlines (Fig. 1C). This implies that variation in traits and its observed relationship with depth, temperature and seasonality in temperature is consistent across regions with markedly different species compositions. The absence of a latitudinal gradient in sampling site scores in the North-East Pacific is likely due to the considerably weaker gradient in temperature (Supplementary Fig. 1) caused by the southward flowing California current bringing cold water along the entire Pacific U.S. coastline^10^. The structuring effect of temperature, seasonality and depth is confirmed by the fourth-corner analysis displaying significant correlations with growth, age at maturity and lifespan (Fig. 2).

**Figure 3:**
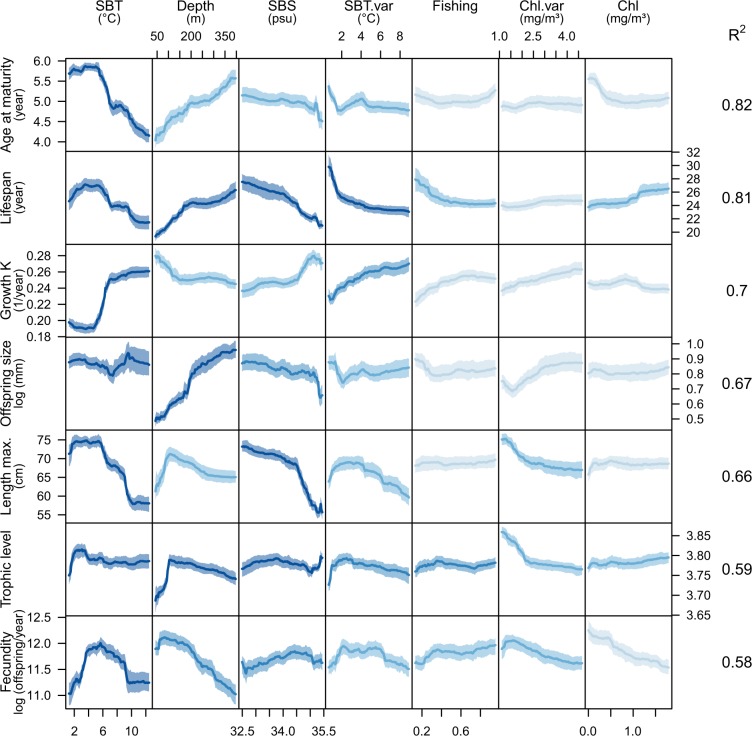
Trait-environment relationships based on random forests, modelling community-weighted mean traits. Seven different models were computed, one for each trait, shown in rows. Colours indicate the importance of predictor variables for each model (in columns), ranging from low importance (light blue) to high importance (dark blue). Response curves were computed for predictor values falling in between the 5^th^ and 95^th^ quantile. The proportion of variance explained by each model (R^2^) is shown on the right side of the plot. The confidence interval was computed based on the 100 RFs with a different training set each time. SBT: sea bottom temperature, SBS: sea bottom salinity, SBT.var: seasonality in sea bottom temperature, Chl.var: seasonality in chlorophyll *a* concentration, Chl: chlorophyll *a* concentration.

The shape of the trait-environment relationships was investigated using RFs by modelling the community-weighted mean (CWM) traits (i.e., the average trait value in a grid cell weighted by species abundances; Supplementary Fig. 2) against the set of environmental variables and fishing pressure. Consistent with the RLQ and fourth-corner analysis, the RFs identify temperature and depth as the best predictors of the spatial variation in traits (Fig. 3). Our suite of methods also demonstrates that fishing pressure is a poor predictor of the spatial distribution of species and traits, at least on the continental scale considered in this study. However, fishing has been shown to affect fish community dynamics^11,12^, especially by targeting large-sized species that are particularly vulnerable to fishing^13^. Consequently, the long history of fishing and high fishing pressure during the last few decades in the studied area may already have impacted the trait composition of the fish communities analysed here. Hence, temporal analyses using longer time scales are needed to further investigate effects of fishing on the trait composition of marine fish communities^11,14^.

The derived response curves reveal several non-linear trait-environment relationships, such as the abrupt increase in the growth coefficient K at intermediate temperatures and the dome-shaped relationships of body length and lifespan with temperature (Fig. 3). The latter can be explained by the presence of Arctic fish species in the coldest environments, which just like sub-tropical fish, tend to have faster life histories than boreal species, thereby possibly allowing them to cope with the extreme climate in the Arctic^15^. Further research and more data from areas with extreme low or high temperatures are necessary to study the trait composition of marine fish communities across the full range of possible temperatures.

Our suite of methods consistently reveal the same key response traits and drivers, showing that fish communities in warm areas and/or at shallow depths are dominated by species characterized by rapid growth, early maturation and short lifespan. Conversely, fish communities in colder, deeper and more seasonally stable environments are primarily composed of slow-growing, late-maturing and long-lived fish species. Our findings are consistent with the classification of organisms, including marine fish^16,17^, along a “fast-slow continuum” that ranks species according to their life-history traits from early-maturing, short-living and fast-growing to late-maturing, long-living and slow-growing^18–20^. The underlying mechanisms explaining this continuum have primarily been related to metabolic theory showing that higher temperatures accelerate metabolic rates but also growth, maturation and mortality (e.g., due to predation, parasitism)^17,21,22^. Environments with high mortality select for fast growth, early reproduction and short lifespan. Conversely, individuals experiencing lower temperatures have lower metabolic rates, but also lower mortality^21,22^. Hence, both mechanisms lead to a slow life history in cold environments. Our results empirically confirm experimental and modelling studies on fish^23–27^ by documenting a strong spatial association between temperature and the key response traits, namely growth, maturation and lifespan.

In addition to the effects of temperature, seasonality can affect fish life-history strategies^28–30^. For instance, opportunistic species, maximizing population growth rates by early maturation and short lifespan, thrive in highly variable environments, since their fast lifestyle allows them to quickly respond to environmental fluctuations^28,29^. Accordingly, we find traits at the fast end of the continuum primarily in shallow and highly seasonal waters.

Our results showing a fast-slow continuum of fish life histories along thermal and depth gradients were consistent when repeating the analysis across spatial scales (Supplementary Fig. 3). Although some differences in trait-environment relationships were observed when splitting the data and RLQ analysis into different depth strata or coastlines (Supplementary Figs. 4, 5), the RFs were still able to identify and incorporate several of these regional differences (Supplementary Figs. 6, 7). This highlights the importance of studying trait biogeography at multiple spatial scales. While individual, regional studies may identify local drivers of trait variation, they may fail to identify global drivers operating at significantly larger geographical scales.

After having identified the key trait-environment relationships and checked their consistency across spatial scales and ecosystems with different species compositions, we demonstrate the usefulness and applied aspects of our trait-based approach by projecting the spatial distribution of fish traits based on the RFs. To avoid projecting outside the range of observed conditions, we restricted projections to subtropical, temperate and Arctic seas of the Northern hemisphere (>20°N) that fall within the observed range of environmental conditions of the original training dataset. The projected distributions of the CWM traits of age at maturity, lifespan and the growth coefficient K reveal the presence of the fast-slow continuum following latitudinal and coastal-offshore gradients across all coastlines (Supplementary Fig. 8). Although latitudinal variation has been shown for body length at the coarse scale of Large Marine Ecosystems^31^, our projections indicate that length, as well as other traits, also vary at a much finer spatial scale due to local environmental variations (Supplementary Figs. 1, 8, 9). However, since our data are mainly based on demersal and bentho-pelagic species from temperate waters and habitats that permit bottom trawling, we caution that our projections are not reliable in areas with inherently different habitats (e.g., coral reefs, deep-sea or pelagic ecosystems). Hence, future studies on trait distributions of marine fish should strive to collect and include other local and regional fish surveys from a variety of habitats, as well as from the Southern hemisphere, in order to test if the trait-environment relationships and projections presented in this study hold for marine fish communities in general.

Our trait-based approach has the great benefit of comparing communities with completely different species compositions (in our case the North Atlantic and North-East Pacific), but the approach comes with some challenges in terms of data needs. In particular, a large amount of trait information is required that may not be readily available for all species, or not estimated with adequate precision. For example, trophic levels are highly variable^32^ and hard to estimate, especially for species with limited diet information. The high uncertainty in trophic level estimates might, or at least partly, explain the relatively low score of trophic level in the RLQ analysis and its low predictability based on RFs. Additionally, a lack of knowledge of intraspecific trait variation can lead to an underestimation of the true variation in traits. This is particularly true for fish that undergo large ontogenetic shifts in traits (e.g., in terms of size, diet and fecundity) throughout their life. Consequently, future trait-based studies should strive to better account for intraspecific variation in traits.

Policy makers, managers and the general public are rightly concerned that marine ecosystems and the services that they supply are under threat from multiple pressures, including overexploitation and climate change^33^. Information is needed on what the effects and relative importance of these pressures are, how they interact and how management can adapt to future changes. Our trait-based study provides key insights into the underlying environmental drivers that structure marine fish communities by revealing a pronounced fast-slow continuum of fish life-history traits along environmental gradients. Furthermore, when substituting our results in space for time^34^, they indicate that global warming will lead to an increasing dominance and geographical expansion of fast-growing, early-maturing and short-lived species. This will likely cause a pronounced restructuring of marine fish communities. Consequently, the fishing industry and fisheries management need to adapt to climate change by seeking to redirect and balance fishing effort to reduce human impacts on species less adapted to warming. Knowledge on key traits and trait-environment relationships can thus readily inform marine ecosystem managers and the industry on the expected changes and vulnerability of fish communities to warming, thereby enabling them to anticipate the ecological and socio-economic consequences of climate change.

## Methods

### Survey data

We collated data from 21 scientific bottom-trawl surveys in the North Atlantic and North-East Pacific (Supplementary Table 1). We selected the period 2005 to 2015 in order to have a similar temporal coverage and a consistent sampling period across surveys. Although gears and sampling protocols vary between surveys, they all use bottom trawls and identify catches at species level whenever possible. Abundance data were standardized according to the duration or swept area of the tow, depending on which information on the tows was provided with the survey. Abundances were log-transformed to reduce the influence of outliers, followed by a conversion to relative abundances by dividing the abundance of each species by the total abundance of all species at each sampling site. Using relative abundances allowed for a standardized comparison of sites and areas with different sampling efforts and units. We verified and updated the taxonomy of reported taxa with the World Register of Marine Species^35^ and discarded all non-fish taxa by keeping only organisms from the following classes: Actinopterygii, Elasmobranchii, Holocephali, Myxini and Petromyzonti. The two largest classes are the bony fish (Actinopterygii) and elasmobranchs (Elasmobranchii). The Holocephali are cartilaginous and belong to the same order as the elasmobranchs, whereas the Myxini (hagfish) and Petromyzonti (lampreys) are also vertebrates, but have no vertebral column or jaw, respectively. Finally, we only kept taxa that had been recorded at the family, genus or species level. Our dataset consisted of 77,824 samples, recording the abundance of 1,889 different taxa (1,583 taxa identified at species level, 203 at genus level and 103 at family level).

### Trait data

To broadly represent the life history and ecology of fish in terms of their feeding, growth, survival and reproduction we selected seven commonly used traits^17,29^: maximum body length (cm), trophic level, fecundity (number of offspring produced by a female per year), offspring size (egg diameter, length of egg case or length of pup in mm), age at maturity (year), lifespan (year) and the von Bertalanffy growth coefficient K (1/year) as a proxy for individual growth rate. Trait information was extracted from a publicly available dataset on marine fish traits^36^, for which most trait values were sourced from FishBase^37^ and collected at the level of Large Marine Ecosystems (LMEs) and FAO fishing areas in order to account for intraspecific variation in species traits across areas. No such area-specific trait values were available for trophic level and offspring size. Gaps were filled in with values from primary literature or genus- or family-averaged values. This led to 429 out of 1,889 taxa for which area-specific values were available for at least one trait. We checked the pairwise correlation between traits, and all the Pearson correlations were below 0.7 (Supplementary Fig. 11). Trait values for fecundity and offspring size were log-transformed to reduce the influence of outliers.

### Environmental and fishing pressure data

We selected seven variables representing hydrography, habitat, food availability and anthropogenic pressure, which are known to affect the distribution of fish species^38–40^. Monthly sea bottom temperature (SBT in °C) and sea bottom salinity (SBS in psu) from 2004 to 2015 were obtained from the Global Ocean Physics Reanalysis (GLORYSs2v4) with a spatial resolution of 0.25°^41^. Chlorophyll *a* concentration (Chl in mg/m^3^) served as a proxy for primary production and food availability. Data were downloaded from the GlobColour database^42^ with a spatial resolution of approximatively 4 km. To ensure a spatio-temporal match with the biotic data, we extracted for each unique sample the corresponding monthly values of SBS, SBT and Chl. Additionally, we calculated the annual range of SBT and Chl (maximum value minus minimum value) within the past twelve months of each sample to represent seasonality in terms of temperature (SBT.var) and Chl (Chl.var). Depth was measured during the surveys and provided with the survey data. If unavailable or incorrect (<5% of the stations), we used depth values from the General Bathymetric Chart of the Oceans (GEBCO 2014 grid)^43^. As a measure of anthropogenic pressure, we used the cumulative demersal fishing pressure in 2013, which was estimated globally at a 1 km^2^ spatial resolution^44^. We summed the demersal fishing pressure estimates from both destructive and non-destructive trawling and with both high and low by-catch. We checked the pairwise correlation between environmental variables and fishing pressure, and all Pearson correlations were below 0.7 (Supplementary Fig. 12), although with a notable high correlation between SBT and SBT.var.

### Overview of methods

To assess trait-environment relationships we collated the three datasets: survey data on fish species relative abundances, trait information, and environmental and fishing pressure data. We removed taxa with incomplete trait information (removed taxa represented <5% of total abundance) and sampling sites that lacked data on the environment or fishing pressure, as no missing values are allowed by the methodological approach. This led to a final dataset of 72,258 samples and 1,464 taxa (325 species, 21 genus-level taxa and 29 family-level taxa with area-specific trait information for at least one trait, and 931 species, 121 genus-level taxa and 37 family-level taxa with no area-specific trait information). The datasets were analysed using three complementary approaches^45^. First, two species-based analyses, RLQ and fourth-corner analysis^9^, were used to summarize the structure among the three datasets to infer key traits and trait-environment relationships based on fish species relative abundances. Then, a community-based approach used the relative abundance data and trait information to calculate community-weighted mean (CWM) traits per grid cell. These CWM traits were then modelled against the environmental variables and fishing pressure by constructing random forests, followed by projecting spatial trait distributions using the constructed models.

### Species-based analyses

The fourth-corner and RLQ analyses are two complementary approaches^9^ that are based on a species-occurrence or species-abundance matrix (L), a species-trait matrix (Q) and an environment-sites matrix (R). The fourth-corner analysis tests pairwise relationships between traits and environmental variables, whereas RLQ considers the inter-correlation of traits and environmental variables. The RLQ analysis is a multivariate analysis and an extension of co-inertia analysis, which is an ordination method exploring the link between two matrices. RLQ analysis explores the relationships between the three matrices R, L and Q, and the method and its mathematical background are described in detail by Dolédec *et al*.^46^ and Dray *et al*.^9^. First, we performed a correspondence analysis on the relative abundance matrix L^47,48^ and principal component analyses on matrices Q and R by using the scores of the sampling sites and species from the previous correspondence analysis on matrix L as weight of the rows. The RLQ analysis combines these three separate analyses and maximizes the cross-covariance between the environmental and trait ordinations, resulting in a co-structure between the three matrices, which is quantified through so-called RLQ axes. The associations between species, traits and environmental variables along the RLQ axes represent the best compromise between traits and environmental variables through species abundances^9^. Variables that have the highest positive or most negative score on the RLQ axes are contributing the most to the observed spatial patterns and trait-environment relationships, while variables with a score close to 0 do not contribute to the observed relationships. Similar to other multivariate analyses, the sign of the scores on the RLQ axes does not have a unit, and multiplying all scores by −1 would not change the interpretation of the results.

We tested the sensitivity of the RLQ analysis to different factors. First, we tested for potential scale-dependency between traits and environment by spatially aggregating sampling sites into grid cells of 0.25°, 0.5°, 1° and 2.5° resolution. For this we calculated the average relative abundance of each species per grid cell over all samples, as well as the average environmental conditions and fishing pressure per grid cell. We also divided our dataset into three different regions, following the coastlines of the North-East Atlantic, North-West Atlantic and North-East Pacific, to test the validity of the RLQ analysis in these three areas. Finally, we divided our dataset into four different depth strata (<30 m, 30–150 m, 150–300 m, >300 m), to test the influence of depth on our results.

The fourth-corner method computes a trait-environment correlation matrix (so-called fourth-corner matrix) based on the three matrices R, L and Q^49^. To reduce computation time, sampling sites were aggregated into grid cells of 0.25° by 0.25°. For each grid cell the average relative species abundance and environmental condition were calculated over all sampling sites falling into the grid cell. Then, a permutation test on the abundance matrix L was performed with 5,000 permutations of rows and columns successively. The correlations between the traits and environmental variables were calculated with the randomly permuted L matrix. The actual correlation values were compared to these correlations with permutation to obtain the significance level of the correlations between traits and environment^9^. The resulting p-values were adjusted for multiple testing, following the false discovery rate procedure^50^.

### Community-based approach

Community-weighted means for all seven traits were computed as geometric mean trait values, by weighting the trait values by species abundances in the community^51,52^. Random forests^53^ were applied to model the CWMs of each trait with the six environmental variables and the fishing pressure variable as predictors. RFs do not make any assumptions on the distribution of the data, consider the interactions between variables, and are known to have good predictive skills^54^. To reduce the computational power required for modelling, samples were placed on a 0.25° by 0.25° grid, which was first used to calculate relative species abundances and environmental variables per grid cell, followed by calculating the CWM traits per grid cell (8,022 grid cells in total).

RFs were generated with 100 regression trees per trait, for which two predictor variables were randomly selected for each individual tree. We trained the RFs for each trait on a training dataset containing 75% of the data (6,016 randomly selected grid cells) and selected the best RF over 10 RFs computed based on the R^2^. The R^2^ was calculated from the test dataset (the remaining 25% of the data) as one minus the ratio between the mean squared error of the predicted and observed values divided by the variance of the response variable. We repeated the selection of the training dataset and the optimizations 100 times. This procedure yielded 100 optimized RFs per trait, which were used to compute the average response of the CWM traits and a confidence interval of the response curves. The response curves of the traits to each predictor were presented under average conditions of the other predictors (defined by the median) and were computed for predictor values falling in between the 5^th^ and 95^th^ quantile. Relative importance of predictors was calculated as the relative decrease in accuracy when permuting the values of one predictor at the time. A value of 1 is then given to the predictor with the strongest decrease in accuracy. Similar to the sensitivity tests of the RLQ analysis, we also plotted the response curves by depth stratum and coastline to see how the RFs project the CWM traits for each depth stratum or coastline separately.

### Projecting trait patterns

After having trained the RFs based on the available data, we used the fitted models to project the CWM trait values in subtropical, temperate and Arctic shelf seas above 20°N across the Northern hemisphere. We constructed a 0.25° by 0.25° grid and calculated the SBT.var and Chl.var as well as annually-averaged values of SBT, SBS and Chl, quantified as the mean across the monthly averages between 2005 and 2015 for each grid cell. We also extracted depth and fishing pressure for all grid cells. To avoid predicting outside the range of observed environmental conditions and exclude extreme conditions for which we only have few data points, we restricted the projections to grid cells with observed values within the 0.5^th^ and 99.5^th^ percentile of the original training dataset. We also restricted the projections to continental shelves by predicting within LMEs, which go up to 200 nautical miles out of the coast. Predictions were made with each of the 100 optimized RFs per trait, which were then used to calculate the average prediction per grid cell, as well as the standard deviation. All data analyses were performed in the statistical software R^55^. The RLQ and fourth-corner method are implemented in the ade4 package^56^ and the RFs were computed with the package randomForest^57^.

## Supplementary information

Supplementary Information

## Data Availability

The majority of the European fish survey data are publicly available from the Database for Trawl Surveys (DATRAS) maintained by the International Council for Exploration of the Sea (http://www.ices.dk/marine-data/data-portals/Pages/DATRAS.aspx). The Norwegian fish survey data were made available through the Institute of Marine Research, Norway, and are available from Djupevåg^58^. The Icelandic survey data are not publicly available, but are available on reasonable request from the Marine and Freshwater Research Institute, Iceland. The survey data from southern Greenland are not publicly available and were provided for this study by Heino Fock from the Thünen Institute, Germany. The French Mediterranean survey data are publicly available from IFREMER (http://www.ifremer.fr/SIH-indices-campagnes/). The Spanish survey data are only partially publicly available on DATRAS. The full dataset is available upon request from the Spanish Institute of Oceanography. The North American survey data were accessed through the public GitHub repository ‘trawlData’ created by Ryan Batt: https://github.com/rBatt/trawlData. These data have been made publicly available by the Alaska Fisheries Science Center, Northwest Fisheries Science Center, Gulf States Marine Fisheries Commission, Northeast Fisheries Science Center and Fisheries and Oceans Canada. More information on the surveys can be found in the Supplementary Information. The trait data are publicly available from PANGAEA^36^. Temperature and salinity data were obtained from the Global Ocean Physics Reanalysis with (GLORYSs2v4) and were downloaded from the Copernicus Marine Environment Monitoring Service (http://marine.copernicus.eu/). Chlorophyll *a* concentrations were downloaded from the GlobColour database (http://hermes.acri.fr/). Depth measurements were provided with the survey data described above, and supplemented with data from the General Bathymetric Chart of the Oceans (https://www.gebco.net). Global fishing pressure data were estimated by Halpern *et al*.^44^ and are available from https://knb.ecoinformatics.org/view/doi:10.5063/F19Z92TW. An aggregated version of the data containing information about fish abundances, environment and traits, as well as code to run the analyses are available in the Dryad repository (10.5061/dryad.ttdz08kt8).
