# Supplementary material for: Marine fish traits follow fast-slow continuum across oceans
Source: Sci Rep. 2019 Nov 29;9:17878. doi: 10.1038/s41598-019-53998-2 (PMC6884637; doi:10.1038/s41598-019-53998-2)
Supplement: Supplementary file 1 — Supplementary Information [file 41598_2019_53998_MOESM1_ESM.pdf]

Supplementary Information to

Marine fish traits follow fast-slow continuum across oceans

Esther Beukhof, Romain Frelat, Laurene Pecuchet, Aurore Maureaud, Tim Spaanheden Dencker, Jón Sólmundsson, Antonio Punzon, Raul Primicerio, Manuel Hidalgo, Christian Möllmann and Martin Lindegren

Content

Supplementary Table 1: Survey data overview
Supplementary Figure 1: Maps of environmental variables and fishing pressure
Supplementary Figure 2: Maps of community-weighted mean traits
Supplementary Figure 3: Sensitivity to spatial resolution
Supplementary Figure 4: RLQ analysis per depth stratum
Supplementary Figure 5: RLQ analysis per coastline
Supplementary Figure 6: Random forest response curves by depth stratum
Supplementary Figure 7: Random forest response curves by coastline
Supplementary Figure 8: Projections of community-weighted mean traits
Supplementary Figure 9: Uncertainty of projections
Supplementary Figure 10: Pairwise correlation between traits
Supplementary Figure 11: Pairwise correlation between environmental variables and fishing

Supplementary Table 1: Survey data overview

Supplementary Table 1. Information on the bottom trawl surveys describing the area, months that the survey is running, number of hauls (samples), gear type when known, the unit of fish abundance, minimum and maximum trawl depth, source of the data when publicly available and survey manual. N/A indicates information is not available. (Table continues on the next page.)

Survey	Area	Year	Month	Number of hauls	Gear type	Abundance unit	Depth min (m)	Depth max (m)	Source	Reference
**AI**	Aleutian Islands	2006, 2010, 2012, 2014	Jun.-Aug.	1605	Poly Nor'Eastern trawl	no./ha	30	1210	1	2
**EBS**	Eastern Bering Sea Shelf	2005-2014	Jun.-Aug.	3756	83-112 Eastern trawl	no./ha	20	210	1	2
**EVHOE**	Bay of Biscay & Celtic Sea	2005-2015	Oct.-Dec	1535	GOV 36/47	no./hour	20	560	3	4
**FR-CGFS**	English channel	2005-2015	Sep.-Nov.	1055	GOV 36/47	no./hour	10	80	3	5
**FR-MEDITS**	French Mediterranean coast	2005-2015	May-Jul.	945	GOC 73	no./km^2	20	870	6	7
**GMEX**	Gulf of Mexico	2005-2015	All	8681	Shrimp trawl	no./km^2	0	850	1	8
**GOA**	Gulf of Alaska	2005, 2007, 2009, 2011, 2013	May-Aug.	3700	Poly Nor'Eastern trawl	no./ha	10	980	1	2
**Gre-GFS**	Greenland	2005-2015	Oct.-Nov.	1025	140' bottom trawl with steel bobbins	no./hour	50	1460	N/A	9
**Ice-GFS**	Iceland	2005-2015	Feb.-April	5672	Granton trawl	no./hour	20	540	N/A	10
**IE-IGFS**	Ireland Shelf Sea	2005-2015	Sep.-Dec.	1809	GOV 36/47	no./hour	10	750	3	5
**NEUS**	North East US	2005-2015	Feb.-May; Sep-Dec.	7334	Yankee 36 bottom trawl before 2009, 3-bridle 4-seam bottom trawl after 2009	no./ 0.0384 km^2	10	920	1	11
**NI-GFS**	Irish Sea - Ireland	2005-2015	Oct.-Mar.	883	Rock Hopper	no./hour	20	120	3	5
**NorBTS**	Norwegian Sea, Barents Sea and northern North Sea	2005-2015	All	12423	Multiple gears	no./hour	20	2100	12	13,14
**NS-IBTS**	North Sea	2005-2015	Jan.-Mar.;	7741	GOV 36/47	no./hour	10	420	3	15

			Jun.-Sep.							
**PT-IBTS**	Portugal Shelf Sea	2005-2014	Sep.-Nov.	796	NCT	no./hour	20	960	3	5
**ROCKALL**	Rockall plateau	2005-2015 (not 2010)	Aug.-Sep.	385	GOV 36/47	no./hour	130	460	3	5
**SA**	South East US	2005-2014	Apr.-Nov	6338	SC Falcon Trawl W/O ted - double rigged	no./ha	0	10	1	N/A
**SCS**	Scotian Shelf	2005-2011	Feb-Aug.	3084	Western IIA trawl	no./ha	20	1940	1	16
**SP-NORTH**	North of Spain	2005-2015	Sep.-Oct	1390	Baka trawl 44/60	no./hour	40	810	3	5
**SWC-IBTS**	Scotland Shelf Sea	2005-2015	Nov.-Mars	1072	GOV 36/47	no./hour	20	500	3	5
**WCANN**	West Coast US	2005-2014	May-Oct.	6595	Aberdeen trawl	no./ha	60	1270	1	17
			**TOTAL**	**77824**						

Sources and references

1. Batt, R. trawlData: R package for maintaining and manipulating data from bottom trawl survey and associated data sets. (2015). Available at: https://github.com/rBatt/trawlData.
2. Alaska Fisheries Science Center. Groundfish Assessment Program - Bottom Trawl Surveys. Available at: https://www.afsc.noaa.gov/RACE/groundfish/bottom trawl surveys.php.
3. ICES. Database of Trawl Surveys (DATRAS). Available at: https://datras.ices.dk/Data_products/Download/Download_Data_public.aspx.
4. ICES. The EVHOE survey (France). *ICES Documents*. (1997). Available at: http://www.ices.dk/marine-data/Documents/DATRAS Manuals/EVHOEManual.pdf.
5. ICES. *Manual of the IBTS North Eastern Atlantic Surveys*. *Series of ICES Survey Protocols SISP 15* (2017). doi:10.17895/ices.pub.3519
6. IFREMER. Population and community indices derived from scientific surveys carried out by IFREMER. *Systéme d'Informations Halieutiques* (2018). Available at: http://www.ifremer.fr/SIH-indices-campagnes/.
7. MEDITS Working Group. *International bottom trawl survey in the Mediterranean - Instruction manual, Version 6*. (2012).
8. Gulf States Marine Fisheries Commission. Southeast Area Monitoring and Assessment Program (SEAMAP) - Gulf of Mexico Resource Surveys. (2015). Available at: https://www.gsmfc.org/seamap-gomrs.php.
9. Fock, H. O. Driving-forces for Greenland offshore groundfish assemblages: Interplay of climate, ocean productivity and fisheries. *J. Northwest Atl. Fish. Sci.* **39,** 103–118 (2008).

10. Sólmundsson, J. *et al. Manuals for the Icelandic bottom trawl surveys in spring and autumn*. (2010). Available at: https://www.hafogvatn.is/static/research/files/fjolrit-156pdf.
11. Northeast Fisheries Science Center (NEFSC). Ecosystems Surveys Branch. (2018). Available at: https://www.nefsc.noaa.gov/esb/mainpage/.
12. Djupevåg, O. IMR Bottom trawl data 1980-2017. (2018). doi:10.21335/NMDC-1657305299. Available at: http://metadata.nmdc.no/metadata-api/landingpage/15ce748250a85dda02e6e4362552f0b1..
13. Mjanger, H. *et al. Håndbok for prøvetaking av fisk og krepsdyr*. (Institute of Marine Research, 2017). Bergen, Norway.
14. Mjanger, H., Hestenes, K., Olsen, E., Svendsen, B. V. & De Lange Wenneck, T. *Manual for sampling of fish and crustaceans*. (Institute of Marine Research, 2006). Bergen, Norway.
15. ICES. Manual for the International Bottom Trawl Surveys Revision VIII. Series of ICES Survey Protocols SISP 10 - IBTS IX. (2015).
16. Fisheries and Oceans Canada. Multi-Species Bottom Trawl Surveys (2014). Available at: http://www.inter.dfo-mpo.gc.ca/Maritimes/SABS/popec/mf/Multi-Species.
17. Keller, A. A., Wallace, J. R. & Methot, R. D. *The Northwest Fisheries Science Center's West Coast Groundfish Bottom Trawl Survey: History, Design, and Description*. *NOAA Technical Memorandum NMFS-NWFSC-136* (2017). doi:10.7289/V5/TM-NWFSC-136

Supplementary Figure 1: Maps of environmental variables and fishing pressure

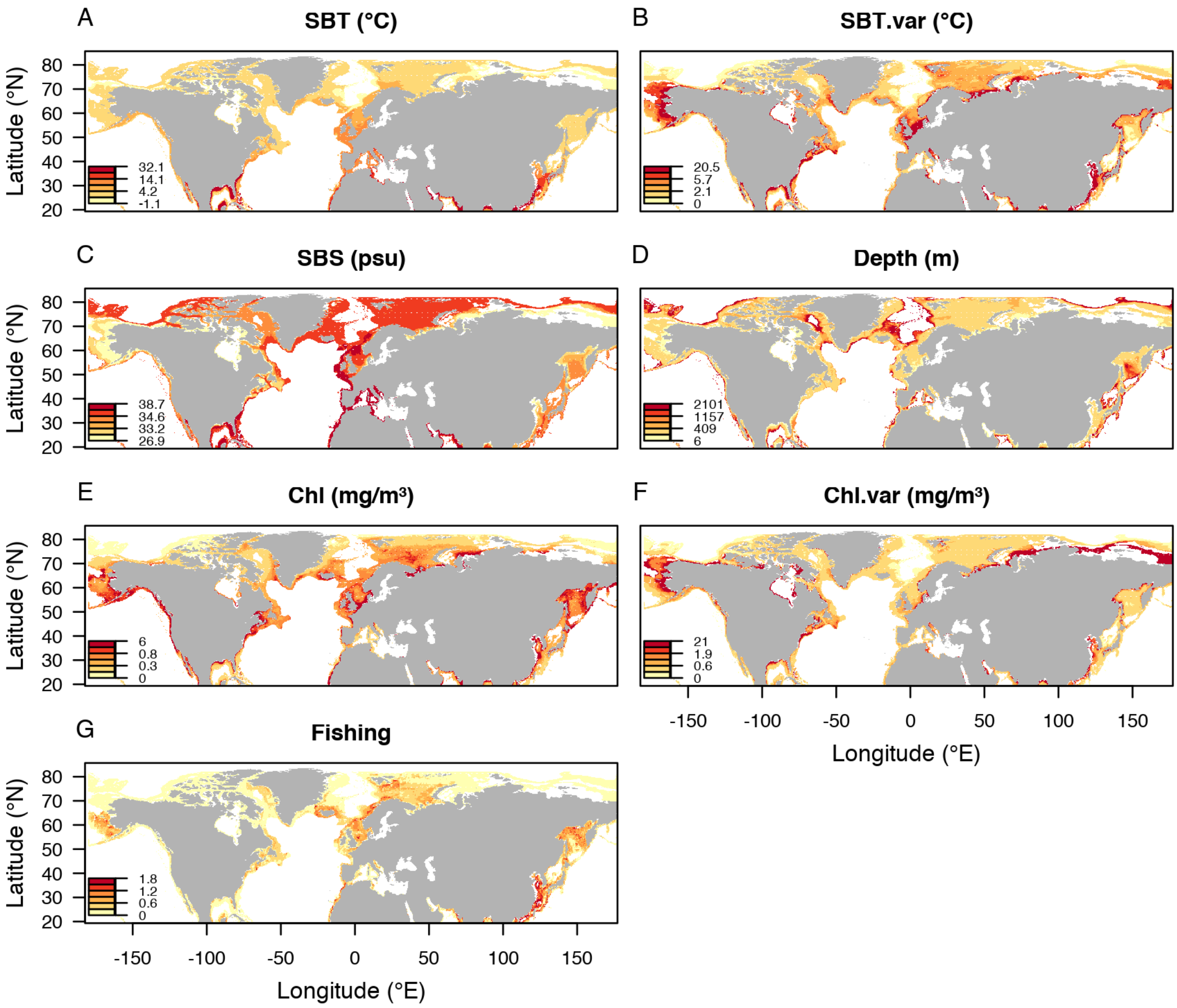

Supplementary Figure 1. Maps of the environmental data and fishing pressure as spatial averages over 2005-2015 for the environmental data and of 2013 for fishing pressure. (A) sea bottom temperature, (B) seasonality in sea bottom temperature, (C) sea bottom salinity, (D) depth, (E) chlorophyll *a* concentration, (F) seasonality in chlorophyll *a* concentration and (G) fishing pressure. Data are plotted on a 0.25° by 0.25° spatial grid.

Supplementary Figure 2: Maps of community-weighted mean traits

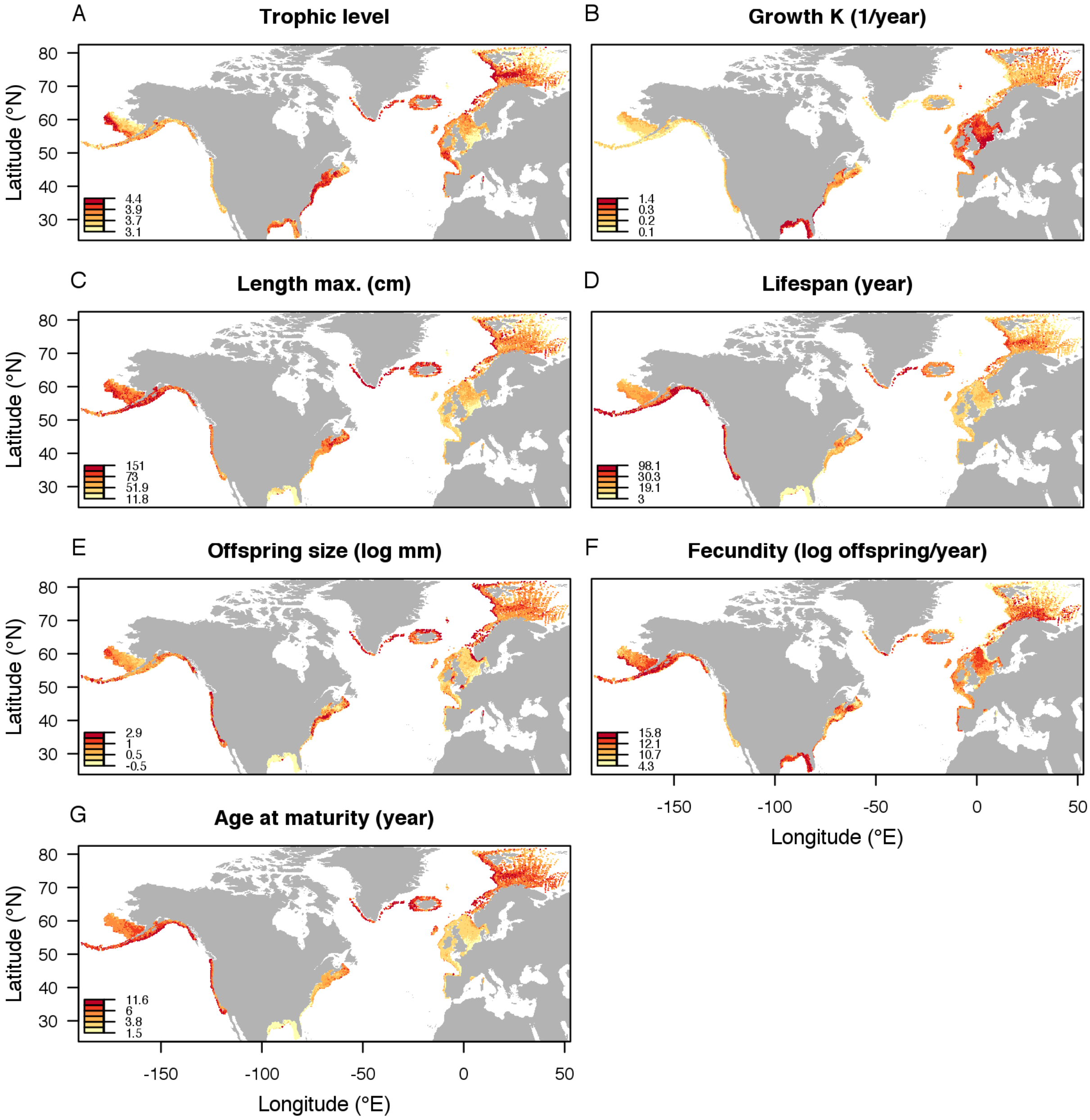

Supplementary Figure 2. Maps of community-weighted mean traits based on spatial averages of species abundances over 2005-2015. (A) trophic level, (B) growth coefficient K, (C) maximum length, (D) lifespan, (E) offspring size (log-transformed), (F) fecundity (log-transformed) and (G) age at maturity. Data are plotted on a 0.25° by 0.25° spatial grid.

Supplementary Figure 3: Sensitivity to spatial resolution

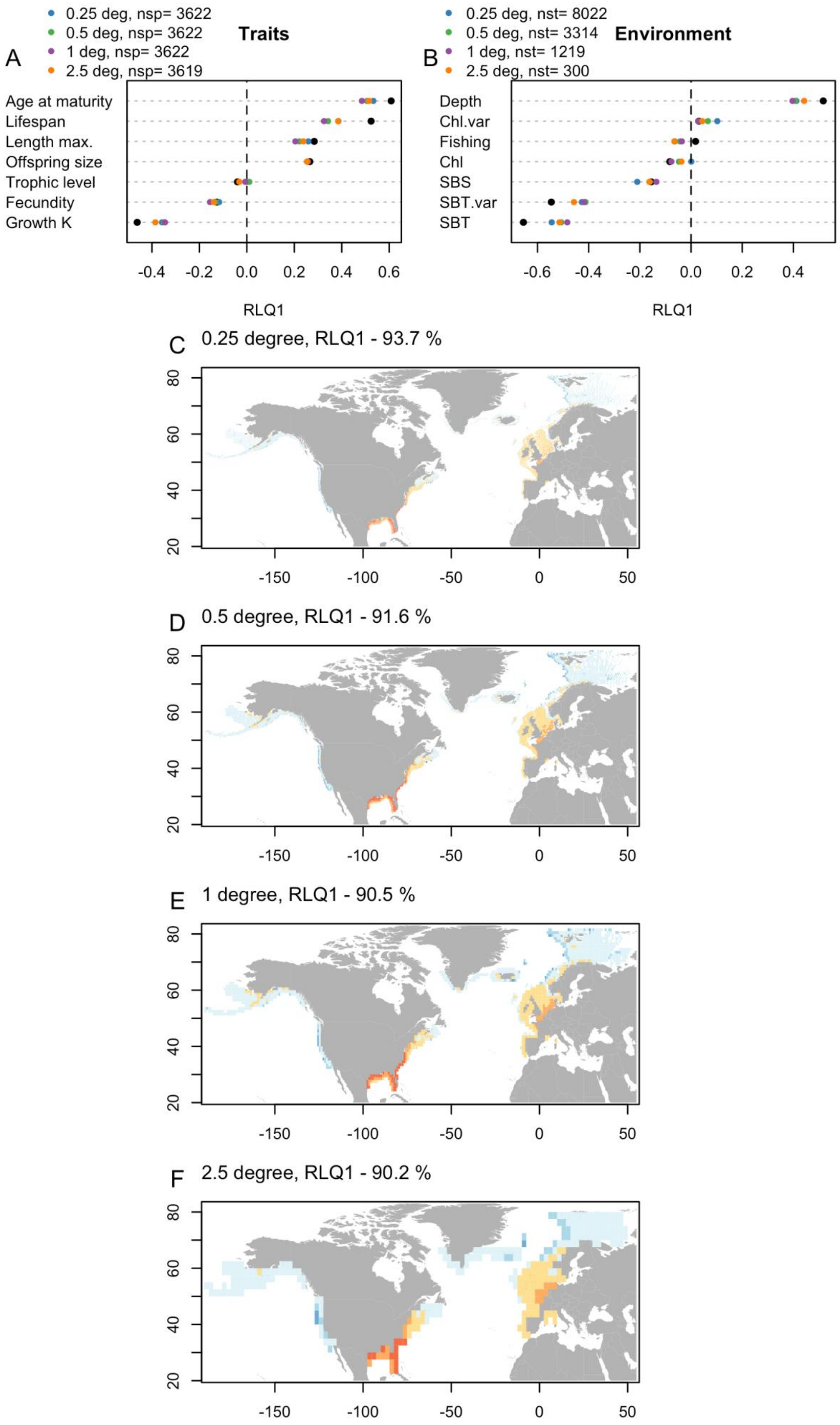

Supplementary Figure 3. RLQ analysis calculated by aggregating samples into a spatial grid with 0.25° (C), 0.5° (D), 1° (E) and 2.5° grid cells (F). The top panel (A,B) shows how traits and environmental variables at each scale are scored along the first RLQ axis (RLQ1) with colours indicating the spatial resolutions and black dots representing the RLQ of the main analysis done by sampling site (i.e., without aggregation into grid cells). The remaining panels show how the grid cells are scored along RLQ1. The percentage above panels C-F represents how much of the cross-covariance between traits and environment has been explained by RLQ1. nsp: number of species, nst: number of grid cells, SBT: sea bottom temperature, SBS: sea bottom salinity, Chl: chlorophyll *a* concentration, SBT.var: seasonality in sea bottom temperature, Chl.var: seasonality in chlorophyll *a* concentration.

Supplementary Figure 4: RLQ analysis per depth stratum

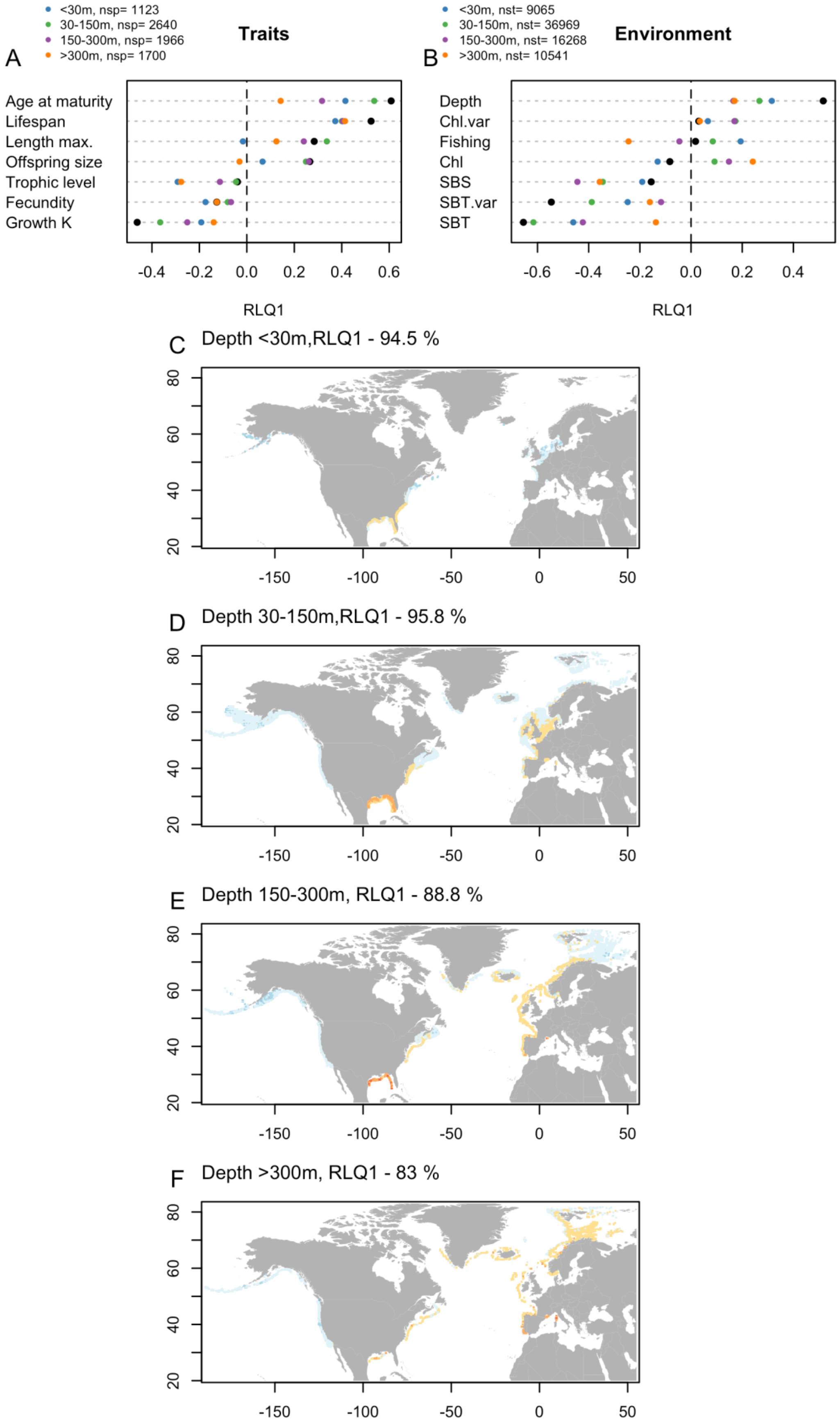

Supplementary Figure 4. RLQ analysis by dividing sampling sites into depth strata: (C) <30 m, (D) 30-150 m, (E) 150-300 m and (F) >300 m. The top panel (A,B) shows how traits and environmental variables are scored along the first RLQ axis (RLQ1) with blue indicating <30 m, green 30-150 m, purple 150-300 m, orange >300 m and black the main analysis based on samples from all depth strata combined. The remaining panels show how the sampling sites are scored along RLQ1. The percentage above panels C-F represents how much of the cross-covariance between traits and environment has been explained by RLQ1. nsp: number of species, nst: number of stations, SBT: sea bottom temperature, SBS: sea bottom salinity, Chl: chlorophyll *a* concentration, SBT.var: seasonality in sea bottom temperature, Chl.var: seasonality in chlorophyll *a* concentration.

Supplementary Figure 5: RLQ analysis per coastline

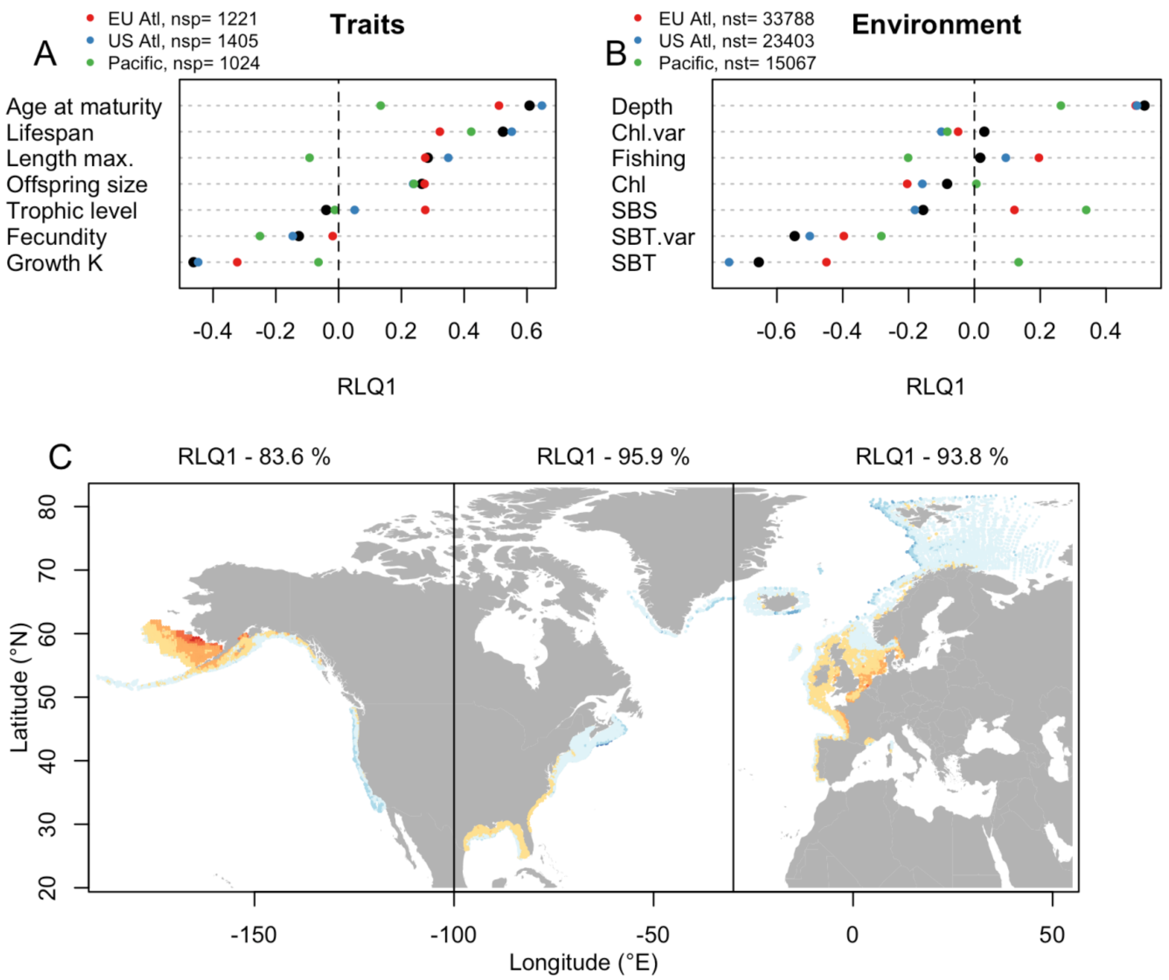

Supplementary Figure 5. RLQ analysis by dividing sampling sites over three coastlines. The top panel (A,B) shows how traits and environmental variables are scored along the first RLQ axis (RLQ1) with red dots indicating North-East Atlantic, blue dots North-West Atlantic, green dots North-East Pacific and black dots the main analysis based on samples from all coastlines combined. The bottom panel shows how the sampling sites are scored along RLQ1. The percentages above the map in panel C indicates how much of the cross-covariance between traits and environment has been explained by RLQ1 for each coastline. nsp: number of species, nst: number of stations, SBT: sea bottom temperature, SBS: sea bottom salinity, Chl: chlorophyll *a* concentration, SBT.var: seasonality in sea bottom temperature, Chl.var: seasonality in chlorophyll *a* concentration.

Supplementary Figure 6: Random forest response curves by depth stratum

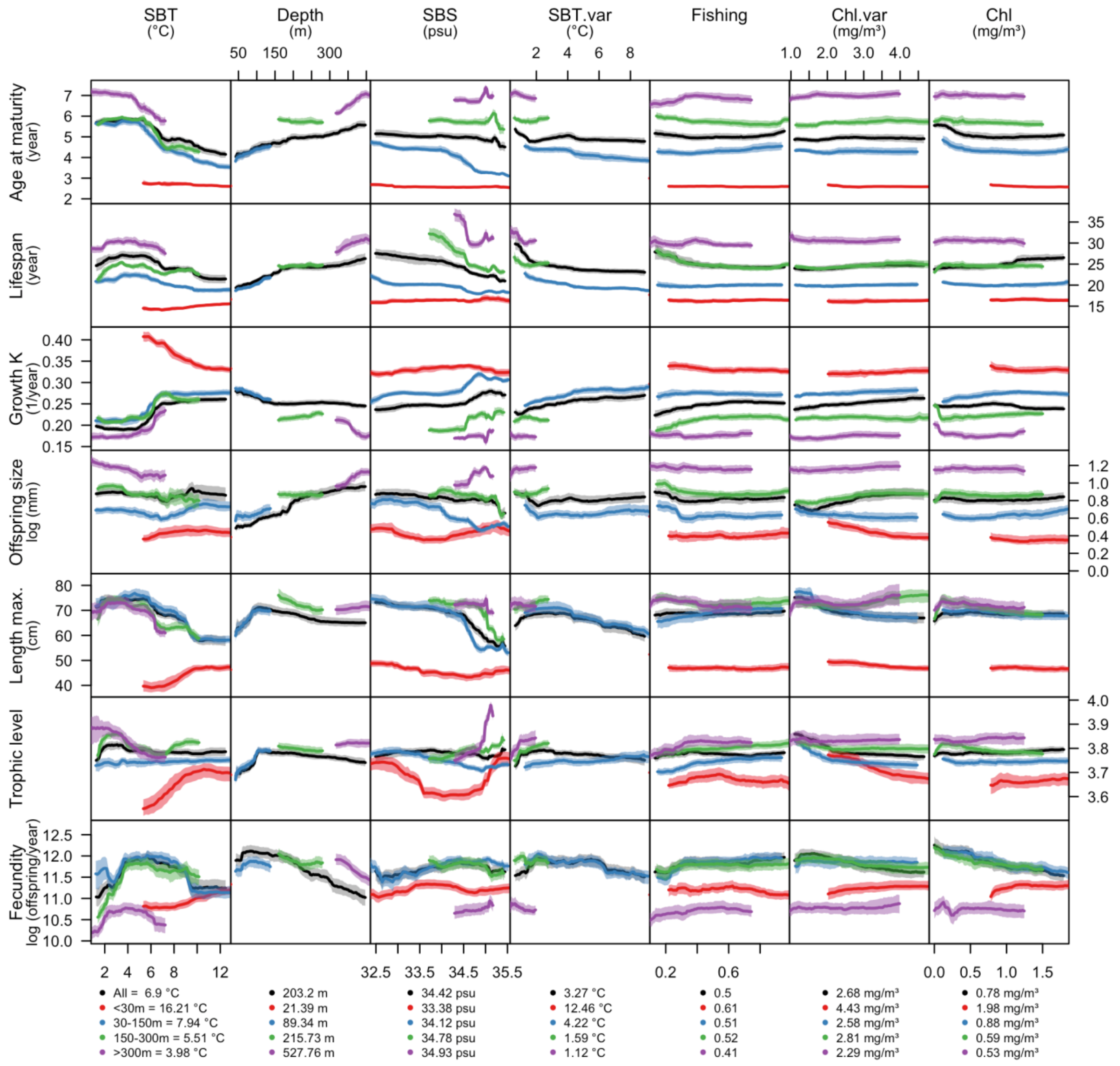

Supplementary Figure 6. Response curves of the random forests plotted by depth stratum, with in red <30 m, blue 30-150 m, green 150-300 m, purple >300 m and black all depth strata combined (as in the main analysis). Each row shows the response of the community-weighted mean traits to the explanatory variables (in columns). The response curves are plotted by keeping all other explanatory variables at their median value (values shown at the bottom of the figure). The red curves are not plotted for SBT.var and depth, because they fall out of the plotting range. SBT: sea bottom temperature, SBS: sea bottom salinity, Chl: chlorophyll *a* concentration, SBT.var: seasonality in sea bottom temperature, Chl.var: seasonality in chlorophyll *a* concentration.

Supplementary Figure 7: Random forest response curves by coastline

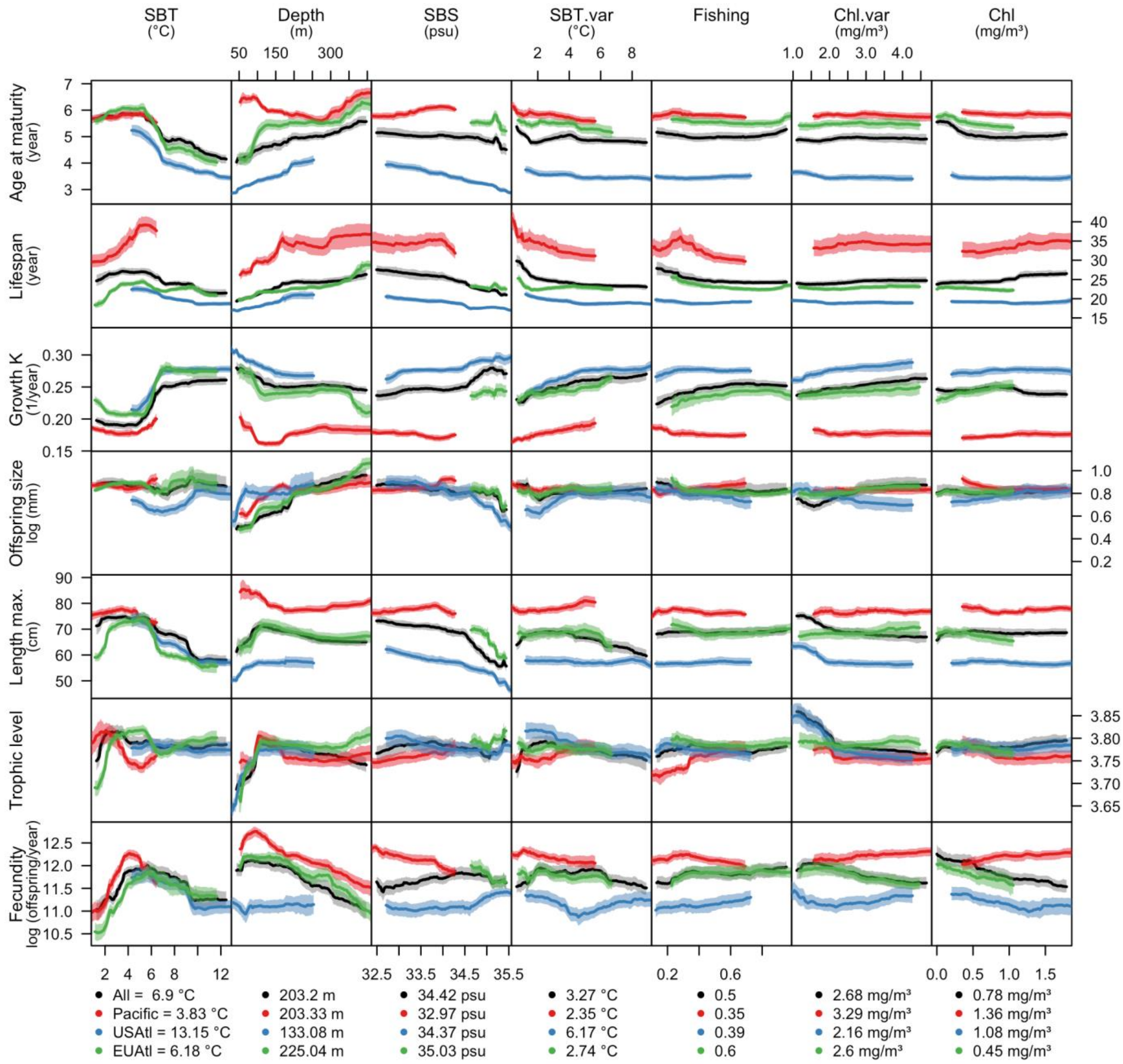

Supplementary Figure 7. Response curves of the random forests plotted per coastline, with in red North-East Pacific, blue North-West Atlantic (USAtl), green North-East Atlantic (EUAtl) and black all coastlines combined (as in the main analysis). Each row shows the response of the community-weighted mean traits to the explanatory variables (in columns). The response curves are plotted by keeping all other explanatory variables at their median value (shown at the bottom of the figure). SBT: sea bottom temperature, SBS: sea bottom salinity, Chl: chlorophyll *a* concentration, SBT.var: seasonality in sea bottom temperature, Chl.var: seasonality in chlorophyll *a* concentration.

Supplementary Figure 8: Projections of community-weighted mean traits

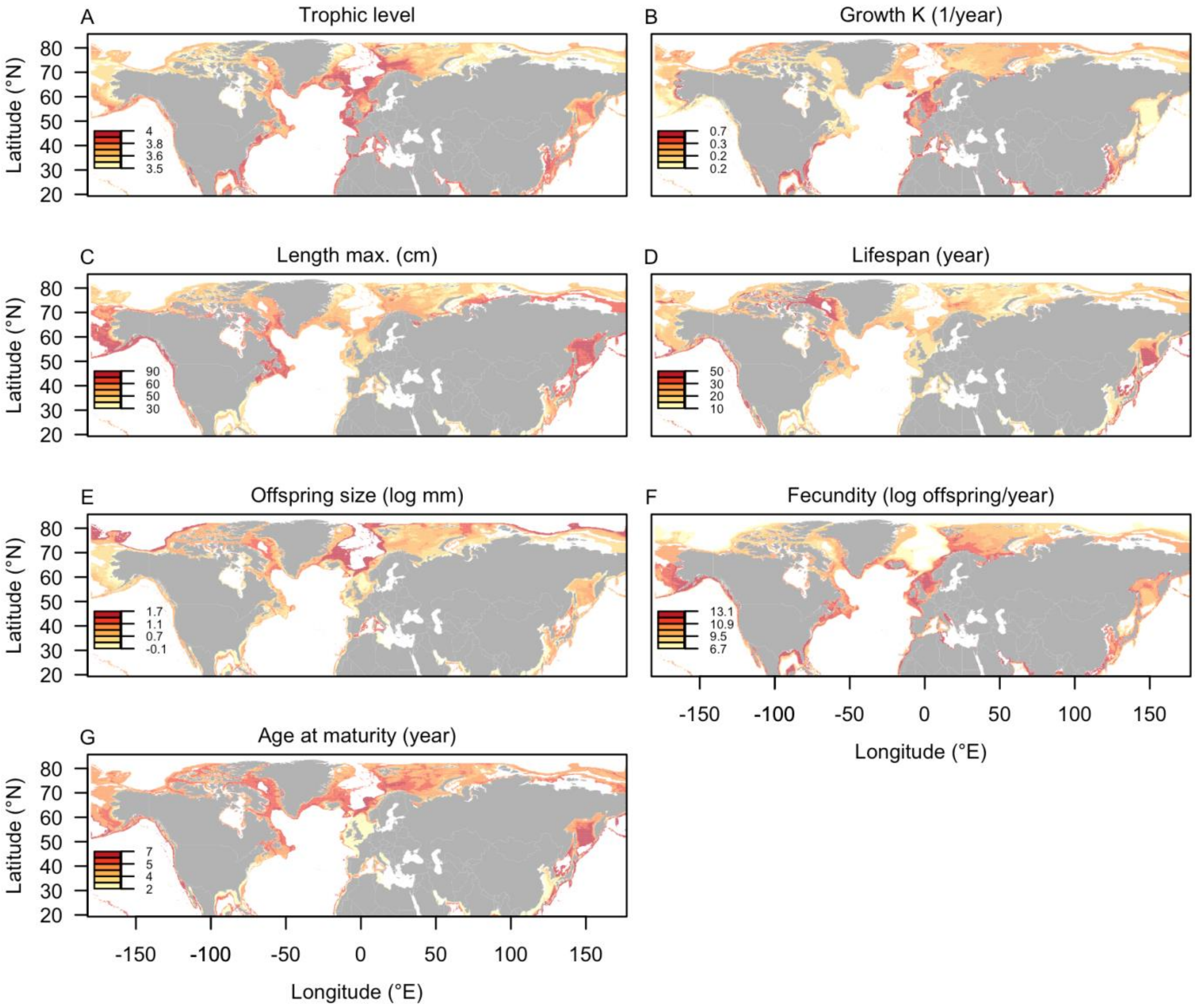

Supplementary Figure 8. Maps of community-weighted mean traits projected by the random forests that modelled community-weighted mean traits against environment and fishing pressure. (A) trophic level, (B) growth coefficient K, (C) maximum length, (D) lifespan, (E) offspring size (log-transformed), (F) fecundity (log-transformed) and (G) age at maturity. Data are plotted on a 0.25° by 0.25° spatial grid and within Large Marine Ecosystems.

Supplementary Figure 9: Uncertainty of projections

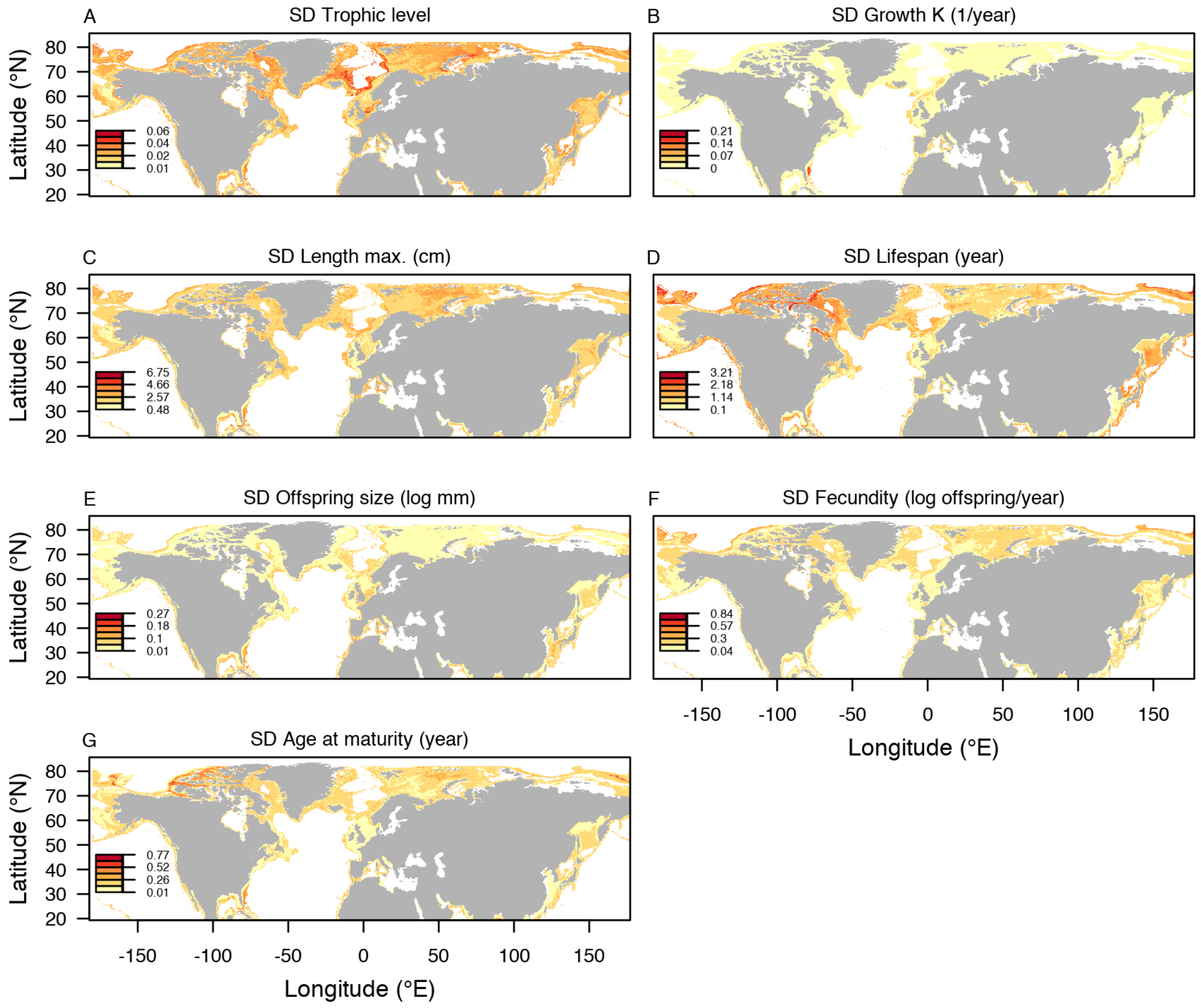

Supplementary Figure 9. Maps of the standard deviations of the community-weighted mean traits projected by the random forests that modelled community-weighted mean traits against environment and fishing pressure. (A) trophic level, (B) growth coefficient K, (C) maximum length, (D) lifespan, (E) offspring size (log-transformed), (F) fecundity (log-transformed) and (G) age at maturity. Data are plotted on a 0.25° by 0.25° spatial grid and within Large Marine Ecosystems.

Supplementary Figure 10: Pairwise correlation between traits

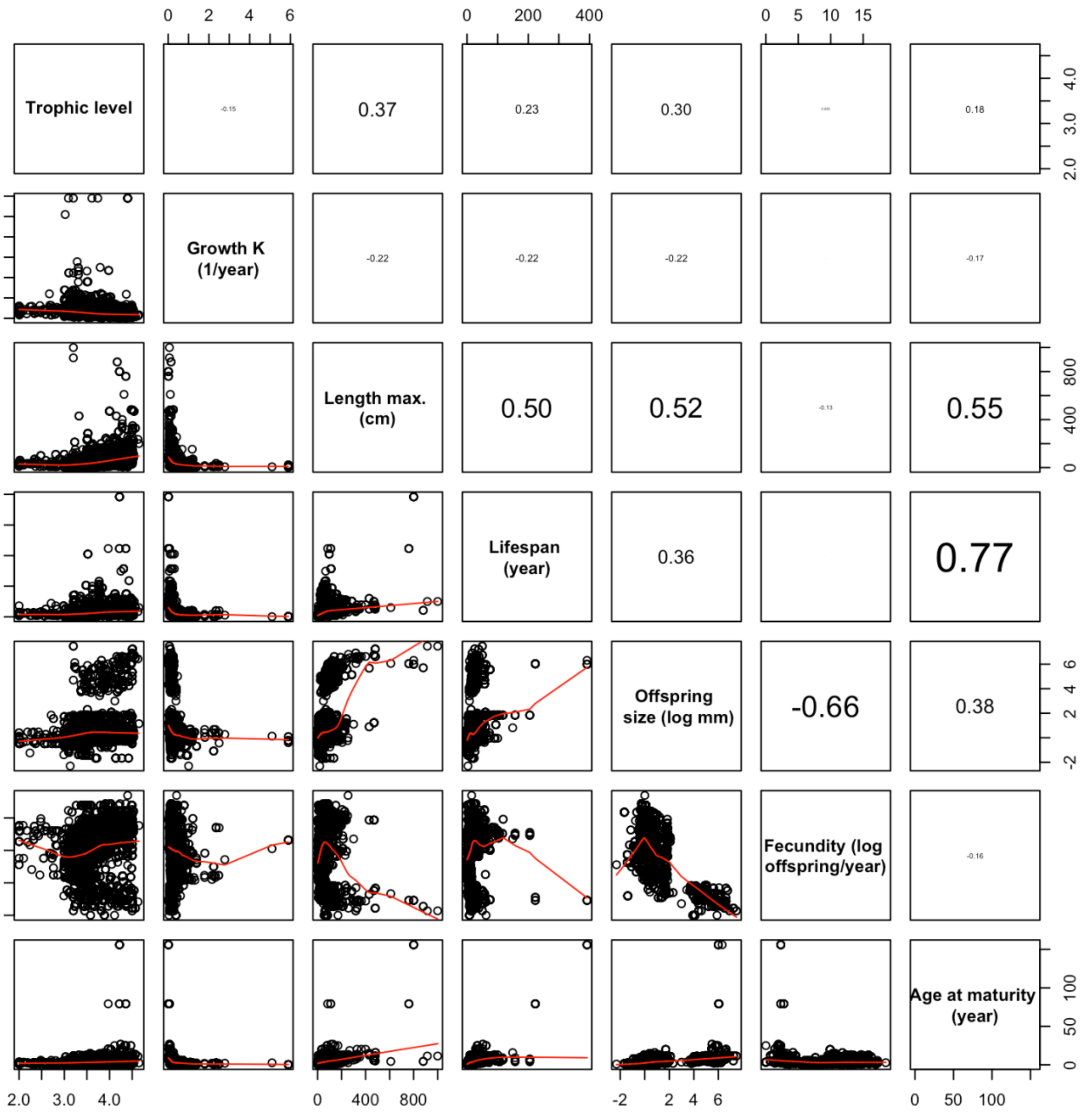

Supplementary Figure 10. Pairwise correlation between the seven selected traits of 1,464 fish taxa. Numbers are Pearson correlation coefficients. Fecundity and offspring size have been log-transformed beforehand.

Supplementary Figure 11: Pairwise correlation between environmental variables and fishing

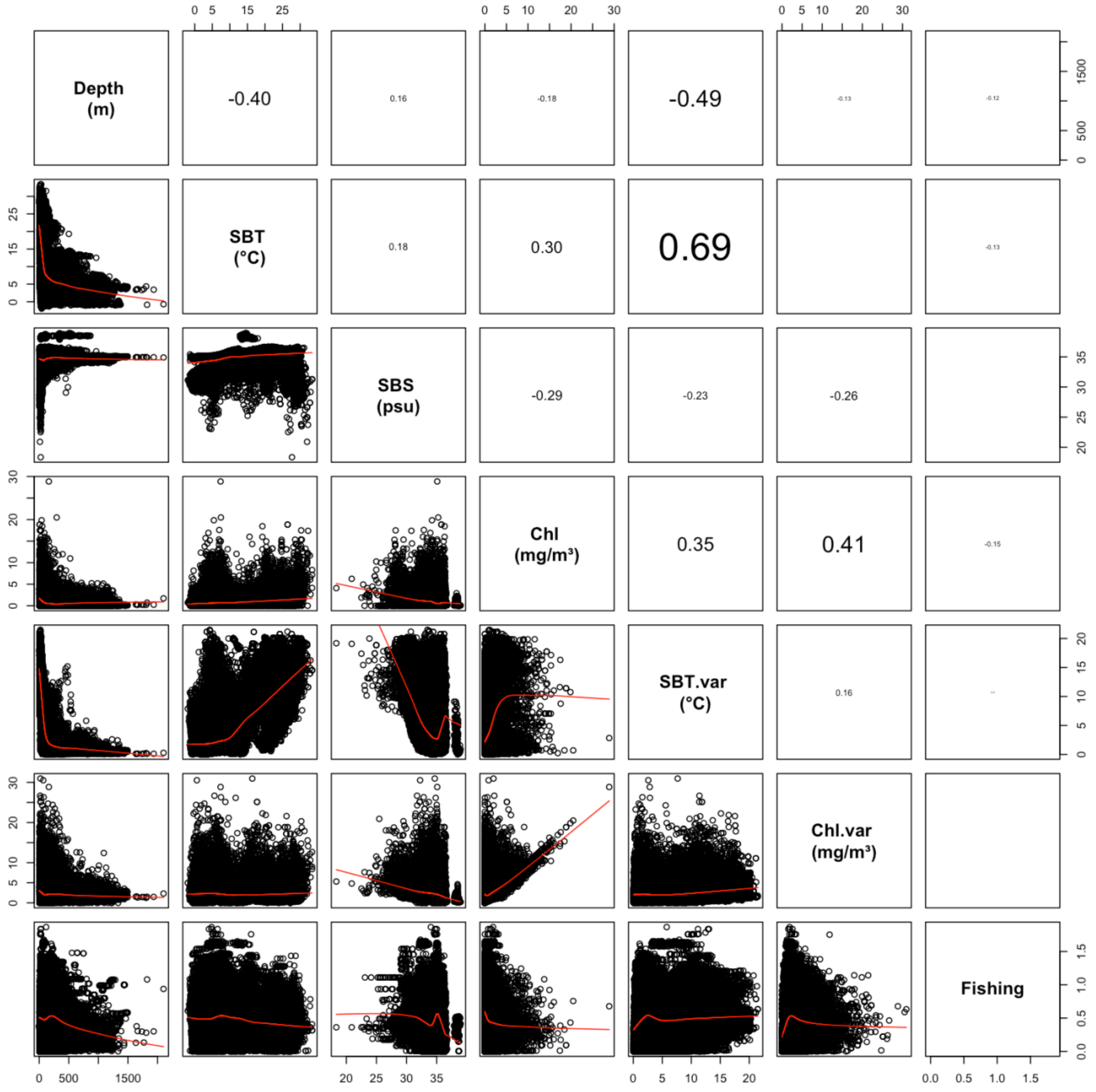

Supplementary Figure 11. Pairwise correlation between the seven selected environmental variables, characterizing 72,258 sampling sites. Numbers are Pearson correlation coefficients. SBT: sea bottom temperature, SBS: sea bottom salinity, Chl: chlorophyll *a* concentration (mg/m^3), SBT.var: seasonality in sea bottom temperature, Chl.var: seasonality in chlorophyll *a* concentration.
